# Interrogating the interactions between myeloid derived suppressor cells and cancer stem cells in glioblastoma

**DOI:** 10.1186/2051-1426-1-S1-P268

**Published:** 2013-11-07

**Authors:** Balint Otvos, James Finke, Michael Vogelbaum, Justin D Lathia

**Affiliations:** 1 Cellular and Molecular Medicine, Cleveland Clinic Foundation, Cleveland, OH, USA; 2 Immunology, Cleveland Clinic Foundation, Cleveland, OH, USA; 3 Brain Tumor and Neuro-Oncology Center, Cleveland Clinic Foundation, Cleveland, OH, USA

## 

**Figure 1:**
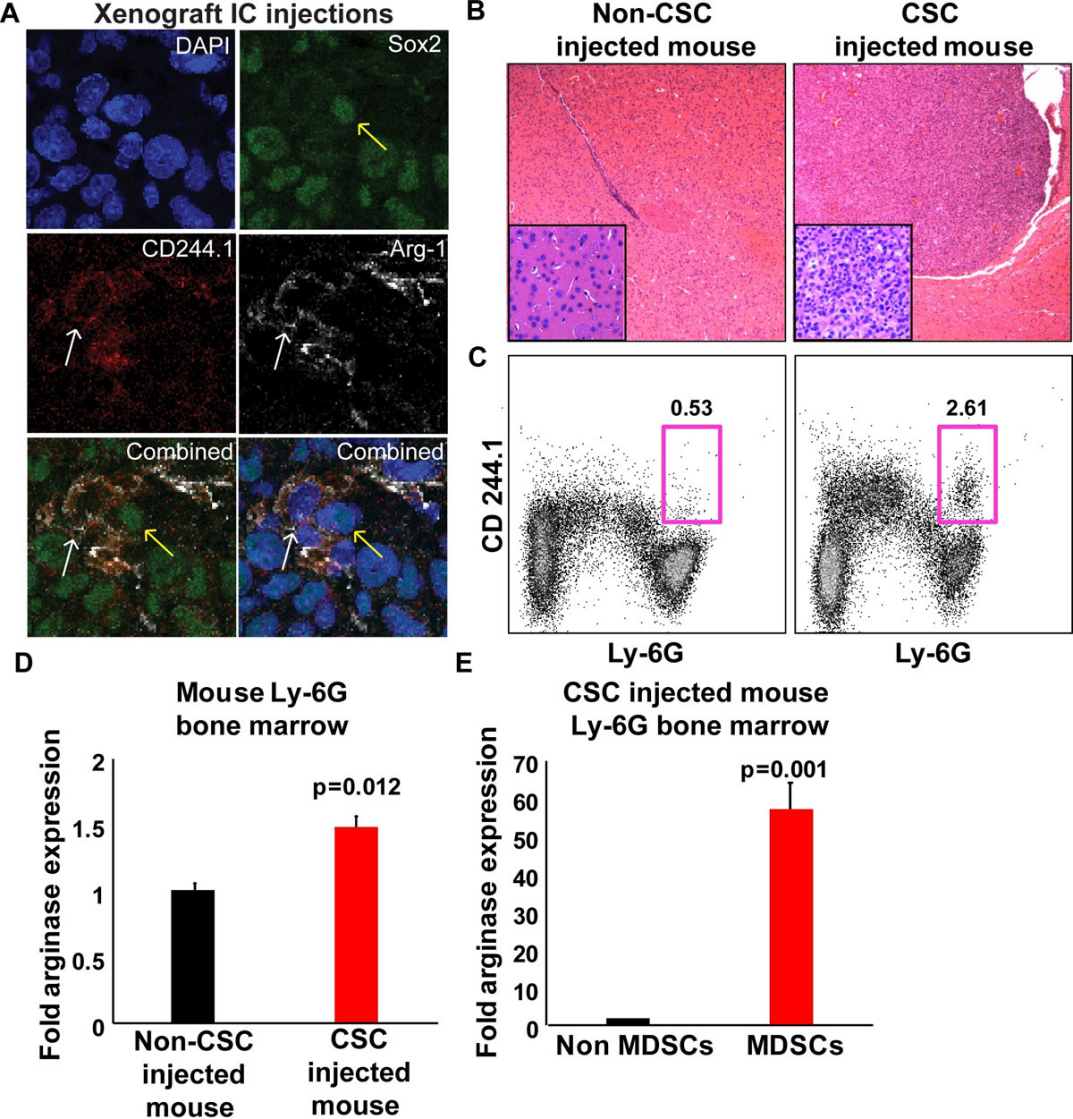
A. MDSCs detected in GBM xenografts by labeling with Sox2, CD244.1, and Arg1. Yellow arrows indicate CSCs; white arrows indicate MDSCs. B. H&E staining of IC xenografts. C. GBM-bearing mice have increased MDSCs by flow cytometry plots (boxed area). D. Arg1 production is increased in GBM mouse marrow. E. MDSCs are responsible for Arg1 production.

Cellular and molecular regulation of the immune system is exquisitely controlled in the brain and is disrupted in neoplasia. Despite accumulation of immune cells in the tumor microenvironment, glioblastoma (GBM) growth persists while the mechanisms suppressing immune function remain largely unknown. Myeloid derived suppressor cells (MDSCs) are a heterogeneous class of immune cells responsible for immunomodulation through the suppression of cytotoxic T-cells. These populations are elevated in the peripheral blood of GBM patients, but their roles within the GBM microenvironment are uncharacterized. Through immunofluorescence analysis, we identified MDSCs within human GBM specimens, suggesting an immunosuppressive phenotype marked by arginase 1 (Arg1) staining. We have also observed co-localization between MDSCs and GBM cancer stem cells (CSCs) in both human tissues and mouse xenografts, leading to the hypothesis that CSCs recruit MDSCs to the tumor microenvironment, promote their survival, and that MDSCs are responsible for the immune-evasive properties of CSCs (Fig. 1A). Intracranial (IC) injections of CSCs into mice have led to GBM formation, expansion of the Arg1-producing MDSCs within the marrow of GBM-bearing mice, and increased levels of MDSCs within the GBM microenvironment (Fig. 1B-E). CSC conditioned media promoted decreased apoptosis and increased Arg1 production of murine bone marrow derived MDSCs. Our results suggest a critical role by which GBM is able to promote immunosuppression through the recruitment of MDSCs and a novel paradigm of immunomodulation by CSCs in GBM.

